# Intravenous albumin for the prevention of hemodynamic instability during sustained low-efficiency dialysis: a randomized controlled feasibility trial (The SAFER-SLED Study)

**DOI:** 10.1186/s13613-021-00962-x

**Published:** 2021-12-13

**Authors:** Edward G. Clark, Lauralyn McIntyre, Irene Watpool, Jennifer W. Y. Kong, Tim Ramsay, Elham Sabri, Mark Canney, Gregory L. Hundemer, Pierre-Antoine Brown, Manish M. Sood, Swapnil Hiremath

**Affiliations:** 1 Division of Nephrology, Department of Medicine, University of Ottawa, Ottawa, ON Canada; 2 Ottawa Hospital Research Institute, Ottawa, ON Canada; 3 Division of Critical Care, Department of Medicine, University of Ottawa, Ottawa, ON Canada; 4 The Ottawa Hospital–Riverside Campus, 1967 Riverside Drive, Ottawa, ON K1H 7W9 Canada

**Keywords:** Renal replacement therapy, Albumin, Acute kidney injury, Hypotension, Ultrafiltration

## Abstract

**Background:**

Hemodynamic instability is a frequent complication of sustained low-efficiency dialysis (SLED) treatments in the ICU. Intravenous hyperoncotic albumin may prevent hypotension and facilitate ultrafiltration. In this feasibility trial, we sought to determine if a future trial, powered to evaluate clinically relevant outcomes, is feasible.

**Methods:**

This single-center, blinded, placebo-controlled, randomized feasibility trial included patients with acute kidney injury who started SLED in the ICU. Patients were randomized to receive 25% albumin versus 0.9% saline (control) as 100 mL boluses at the start and midway through SLED, for up to 10 sessions. The recruitment rate and other feasibility outcomes were determined. Secondary exploratory outcomes included ultrafiltration volumes and metrics of hemodynamic instability.

**Results:**

Sixty patients (271 SLED sessions) were recruited over 10 months. Age and severity of illness were similar between study groups. Most had septic shock and required vasopressor support at baseline. Protocol adherence occurred for 244 sessions (90%); no patients were lost to follow-up; no study-related adverse events were observed; open label albumin use was 9% and 15% in the albumin and saline arms, respectively. Ultrafiltration volumes were not significantly different. Compared to the saline group, the albumin group experienced less hemodynamic instability across all definitions assessed including a smaller absolute decrease in systolic blood pressure (mean difference 10.0 mmHg, 95% confidence interval 5.2–14.8); however, there were significant baseline differences in the groups with respect to vasopressor use prior to SLED sessions (80% vs 61% for albumin and saline groups, respectively).

**Conclusions:**

The efficacy of using hyperoncotic albumin to prevent hemodynamic instability in critically ill patients receiving SLED remains unclear. A larger trial to evaluate its impact in this setting, including evaluating clinically relevant outcomes, is feasible.

*Trial registration* ClinicalTrials.gov (NCT03665311); First Posted: Sept 11th, 2018. https://clinicaltrials.gov/ct2/show/NCT03665311?term=NCT03665311&draw=2&rank=1

**Supplementary Information:**

The online version contains supplementary material available at 10.1186/s13613-021-00962-x.

## Background

Hemodynamic instability is a frequent complication of all forms of renal replacement therapy (RRT) commonly used in the intensive care unit (ICU), including sustained low-efficiency dialysis (SLED) [1]. Hemodynamic instability during RRT (HIRRT) is associated with increased in-hospital mortality [2] and may decrease the likelihood of recovery of kidney function to dialysis-independence [3, 4]. In addition, HIRRT can limit ultrafiltration with RRT and perpetuate fluid overload further worsening mortality and reducing the likelihood of kidney function recovery in patients with acute kidney injury (AKI) [2, 5–7].

Albumin is the primary contributor to intravascular colloid oncotic pressure [8, 9]. Intravenous hyperoncotic albumin may be administered prior to, or during RRT, in order to prevent HIRRT and/or augment ultrafiltration [10]. Theoretically, it works by promoting intravascular plasma refilling from the extravascular compartment to compensate for fluid removal with ultrafiltration. While several small, cross-over trials have reported hemodynamic benefits [11–13], no trial has been powered for clinically relevant outcomes. Furthermore, intravenous albumin is expensive [14] and not without potential harms [10].

Clinical trials are needed to determine if the administration of intravenous albumin to mitigate HIRRT can improve clinically meaningful outcomes. In order to inform the design and assess the feasibility of such a trial, we undertook a feasibility randomized controlled trial (RCT) comparing intravenous 25% albumin to 0.9% saline during SLED sessions initiated in ICU for patients with AKI.

## Methods

### Design, setting and participants

We conducted a single-center, double-blind, placebo-controlled, randomized feasibility trial with two parallel arms as described in a previously published protocol [15]. ICU patients who were ≥ 18 years old and had AKI for which they were planned to receive SLED were included. Exclusion criteria were: SLED for a non-AKI indication (e.g., intoxication); kidney failure receiving dialysis treatment prior to admission; allergy to albumin; pregnancy; and any contraindication or known objection to blood-product transfusions. Patients were recruited from two 30-bed medical–surgical ICUs located at different campuses at our quaternary academic referral center in Ottawa, Canada. This study was prospectively registered (ClinicalTrials.gov Identifier NCT03665311) and approved by the Ottawa Health Sciences Network Research Ethics Board (OHSN-REB) (Protocol ID 20180567-01H) with approval granted for the use of deferred patient consent under circumstances in which the patient was incapacitated and their substitute decision-maker could not be immediately reached. As such, this study has been performed in accordance with the ethical standards lad down in the 1964 Declaration of Helsinki and its later amendments.

The study protocol was amended from the published protocol [15] with respect to the number of patients enrolled. In the absence of similar previous studies to base our estimates [10], the original recruitment target was conservatively determined to be 30 patients within 18 months after starting enrollment. Following a successful initial enrollment period, the decision was taken to amend the protocol to include up to a maximum of 60 patients, principally to facilitate a more definitive assessment of feasibility outcomes than only approximately 15 patients in each arm would have allowed. This amendment was made prior to any data analysis having been performed.

Details regarding the computerized randomization procedure and blinding are available in the previously published protocol [15]. With respect to blinding, briefly, this involved the use of opaque bags to cover glass albumin and saline bottles (with the saline bottles specially prepared for the purpose of the study) and tubing covers.

SLED is the only RRT modality used at our center for hemodynamically unstable patients (i.e., continuous renal replacement therapy (CRRT) is not used). Patients are typically started on, or transitioned to, intermittent hemodialysis in the ICU setting if they are hemodynamically stable. As such, patients are typically treated with SLED at our center if they are receiving vasopressors or were receiving them within the past 12 to 24 h, or are otherwise perceived by the treating team that they would be unlikely to tolerate intermittent hemodialysis. The SLED protocol at our center is for 8-h sessions using a dialysate temperature of 35.5 °C, maximum blood flow rate of 200 mL/min, maximum dialysate flow rate of 300 mL/min and dialyzers with a surface area of 0.6 m^2^. The default settings for the dialysate concentrations of sodium, potassium, bicarbonate and calcium are 140 mmol/L, 4 mmol/L, 34 mmol/L and 1.25 mmol/L, respectively, but were adjusted at the discretion of the treating physicians. Timing of initiation and ultrafiltration goals were ordered entirely at the discretion of the treating physicians (intensivists and nephrologists).

### Intervention

Patients were randomly assigned (1:1 allocation) to receive 100 mL boluses of either 25% albumin or normal saline (0.9%) at the start and then midway (at 4 h) into SLED sessions (200 mL in total, per session). Patients received the same assigned fluid for up to 10 SLED sessions.

### Outcomes

The primary feasibility outcomes were recruitment rate, adherence to the protocol and completeness of follow-up. As per the published protocol [15], if three criteria were all met, progression to the future large trial would be considered feasible. These progression criteria were: (1) recruitment of at least 30 patients within 18 months of enrollment; (2) recruitment of at least 15% of eligible patients during the study period; (3) < 10% of included patients having adverse events related to the interventions (i.e., either albumin or saline). Exploratory outcomes included measures of ultrafiltration, hemodynamic stability during treatment and patient outcomes as detailed in the previously published protocol [15].

### Statistical analysis

All analyses were based on a modified intention-to-treat approach in which only those randomized patients who received at least one SLED treatment after randomization were included for analysis. All data were reported according to the two treatment arms as well as in aggregate using frequency and percentages for binary or categorical variables and means and standard deviations (SD) and/or median and interquartile range (IQR) for continuous variables as appropriate. Due to a skewed distribution, outcomes related to fluid removal were described using median and interquartile ranges for the two study arms and compared between the two groups using non-parametric statistical tests such as Wilcoxon rank sum test. Mixed-effects models were used to compare reductions in systolic blood pressure (SBP) between groups to account for both within-person and between-person variability. Estimates are reported as least squares mean difference with associated 95% confidence intervals. Wilcoxon rank sum test was used to compare ICU length of stay and hospital length of stay between the two groups. ICU and hospital mortality were compared between the two groups using a Chi-square test. The effect was calculated as risk difference with 95% confidence intervals (CIs). P-values were not reported due to the multiple outcomes assessed and the exploratory nature of the analysis. All data manipulations and statistical analyses were performed using Statistical Analysis System, Version 9.4 (SAS Institute Inc., Cary, North Carolina, USA).

This trial is reported in accordance with the Consolidated Standards of Reporting Trials (CONSORT) [16] extension to randomized pilot and feasibility trials [17] (refer to the Additional file 1 for a completed checklist).

## Results

### Characteristics of the study population

**Fig. 1:**
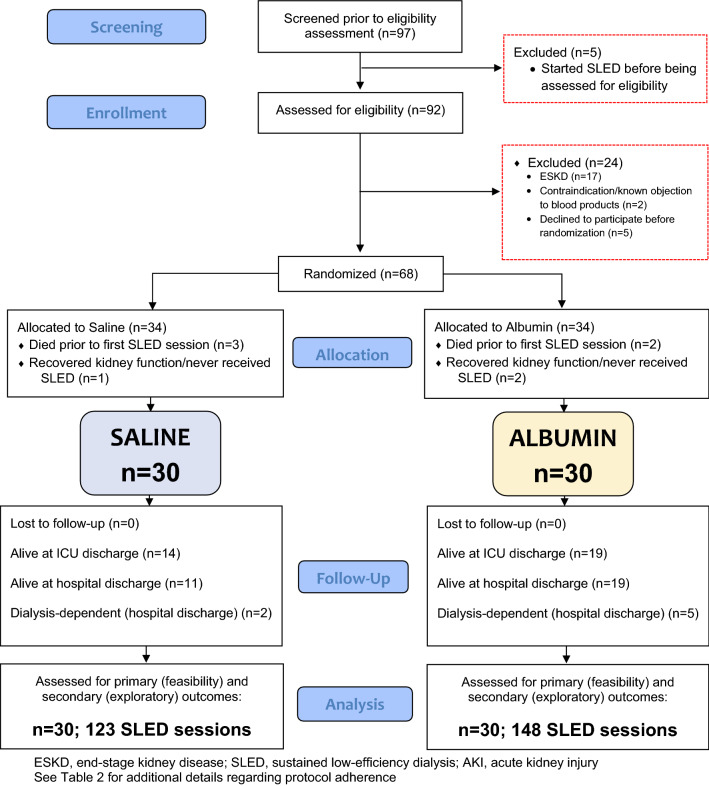
Study flow diagram. ESKD: end-stage kidney disease; SLED: sustained low-efficiency dialysis; AKI: acute kidney injury. See Table 2 for additional details regarding protocol adherence

Between April 1st, 2019 and January 14th, 2020, 97 subjects were screened and a total of 68 were randomized to receive saline or albumin (Fig. 1). Thirty participants in each group received at least one SLED treatment after randomization and were included for analysis (*n* = 60). A total of 271 SLED sessions were performed in this cohort, 123 in the saline group and 148 in the albumin group. The median (IQR) number of SLED sessions per participant was 3 (1, 7) and 4 (3, 8), respectively. Age and APACHE II scores appeared similar between the two study groups. Several other baseline characteristics did not appear balanced between the study groups with more patients in the albumin group initiated on SLED for hyperkalemia. Pulmonary edema and hyperkalemia were the most common indications for the initiation of SLED overall. Mean (SD) pre-SLED serum albumin level (including all SLED sessions) for the saline group was 23 g/L (6.6) g/L and for the albumin group, 26 (7) g/L. Baseline characteristics of included participants are reported in Table 1.

**Table 1:** Baseline characteristics of included patients (prior to first SLED session)

Characteristic	Saline (*n* = 30)	Albumin (*n* = 30)	Overall (*n* = 60)
Age in years, mean (SD)	60 (18)	61 (13)	60 (15)
Female, n (%)	9 (30)	6 (20)	15 (25)
APACHE II total score, mean (SD)	29.9 (8.2)	30.3 (8.1)	30.1 (8)
*SOFA score components prior to first SLED session, median (IQR)*			
Respiration	2 (1,3)	2 (2,3)	2 (1.75,3)
Coagulation	1 (0,2)	1 (0,2)	1 (0,2)
Liver	1 (0,2)	1 (0,2)	1 (0,2)
Cardiovascular	4 (3,4)	4 (3,4)	4 (3,4)
Central nervous system	1 (0,2)	1 (0,1.75)	1 (0,2)
Renal	4 (3.5,4)	4 (4,4)	4 (4,4)
Total	13 (10,15)	12.5 (10.5,14)	12.5 (10,15)
*Comorbid conditions, n (%)*			
Diabetes mellitus	13 (43)	10 (33)	23 (38)
Congestive heart failure	1 (3)	1 (3)	2 (3)
Peripheral vascular disease	3 (10)	6 (20)	9 (15)
Chronic kidney disease	6 (20)	9 (30)	15 (25)
*Primary ICU admission diagnosis, n (%)*			
Septic/distributive shock	19 (63)	22 (73)	41 (68)
Respiratory failure	4 (13)	5 (17)	9 (15)
Other	7 (23)	3 (10)	10 (17)
*Indications for SLED Initiation, n (%)*			
Pulmonary edema	16 (53)	14 (47)	30 (50)
Uremia	1 (3)	0 (0)	1 (2)
Hyperkalemia	8 (27)	17 (57)	25 (42)
Acidosis	13 (43)	14 (47)	27 (45)
Other	2 (7)	1 (3)	3 (5)
*Laboratory parameters prior to first SLED session, mean (SD)*			
Albumin (g/L)	25 (7)	23 (8)	24 (7)
Creatinine (µmol/L)	320 (168)	357 (188)	338 (178)
Urea (mmol/L)	21.6 (11.9)	27.6 (13.5)	24.5 (12.9)
Potassium (mmol/L)	4.6 (0.9)	5.0 (0.9)	4.8 (1.0)
Calcium (mmol/L)	2.02 (0.24)	2.01 (0.26)	2.01 (0.24)
Ionized calcium (mmol/L)	1.06 (0.11)	1.03 (0.1)	1.05 (0.11)
Phosphate (mmol/L)	2.02 (0.83)	2.16 (0.93)	2.09 (0.87)
Lactate (mmol/L)	3.0 (3.2)	4.5 (5.8)	3.7 (4.6)
Hemoglobin (g/L)	90 (19)	91 (21)	91 (20)
White blood cells (×10^9^/L)	14.7 (12.6)	14.8 (11.5)	14.7 (12.0)

SLED: sustained low-efficiency dialysis; *n*: number; SD: standard deviation; APACHE: Acute physiology and chronic health evaluation

### Feasibility outcomes

Enrollment was stopped once the modified goal of enrolling 60 patients who received at least one SLED session was achieved. With respect to the outcome of recruitment rate, the 60 patients were recruited over 10 months. In the cohort overall, both doses of the assigned fluid (either albumin or saline) were administered correctly (according to the study assignment) for 244 of the 271 SLED sessions, thereby achieving 90% adherence to the study protocol. No patients were lost to follow-up.

Of the 97 potentially eligible patients in this 10-month time window, 60 of them were included for analysis (62%) (see Fig. 1). No patients revoked consent after enrollment. Additional feasibility measures are reported in Table 2. No adverse events related to albumin or saline administration were encountered.

**Table 2:** Feasibility measures

Protocol adherence measurements	Saline (*n* = 123 sessions)	Albumin (*n* = 148 sessions)	Overall (*n* = 271 sessions)
Protocol adherence for both doses*, *n* (%)	109 (89)	135 (91)	244 (90)
Assigned albumin or placebo not given at start of SLED, *n* (%)	4 (3)	7 (5)	11 (4)
Assigned albumin or placebo not given after 4 h of SLED, *n* (%)	10 (8)	6 (4)	16 (6)
Both doses of assigned albumin or placebo not given, *n* (%)	0 (0)	0 (0)	0 (0)
Contamination measurements			
Received non-study intravenous albumin during the SLED session, *n* (%)	19 (15)^†^	13 (9)^‡^	32 (12)

SLED: sustained low-efficiency dialysis

*Sessions for which patient correctly received both doses of either albumin or placebo

^†^Nine patients (30%) received non-study intravenous albumin during SLED sessions at least once (for 17 sessions it was given as 100 mL of 25% albumin once, in one session it was given twice, and in one session 250 mL of 5% albumin was given once)

^‡^Six patients (20%) received non-study intravenous albumin during SLED sessions at least once (for 10 sessions it was given as 100 mL of 25% once, in 2 sessions it was given twice, and in one session 250 mL of 5% albumin was given twice)

### Exploratory outcomes

There were no significant differences in the fluid removal between groups. Additional file 1: Table S1 summarizes variables and outcomes related to fluid removal.

**Fig. 2:**
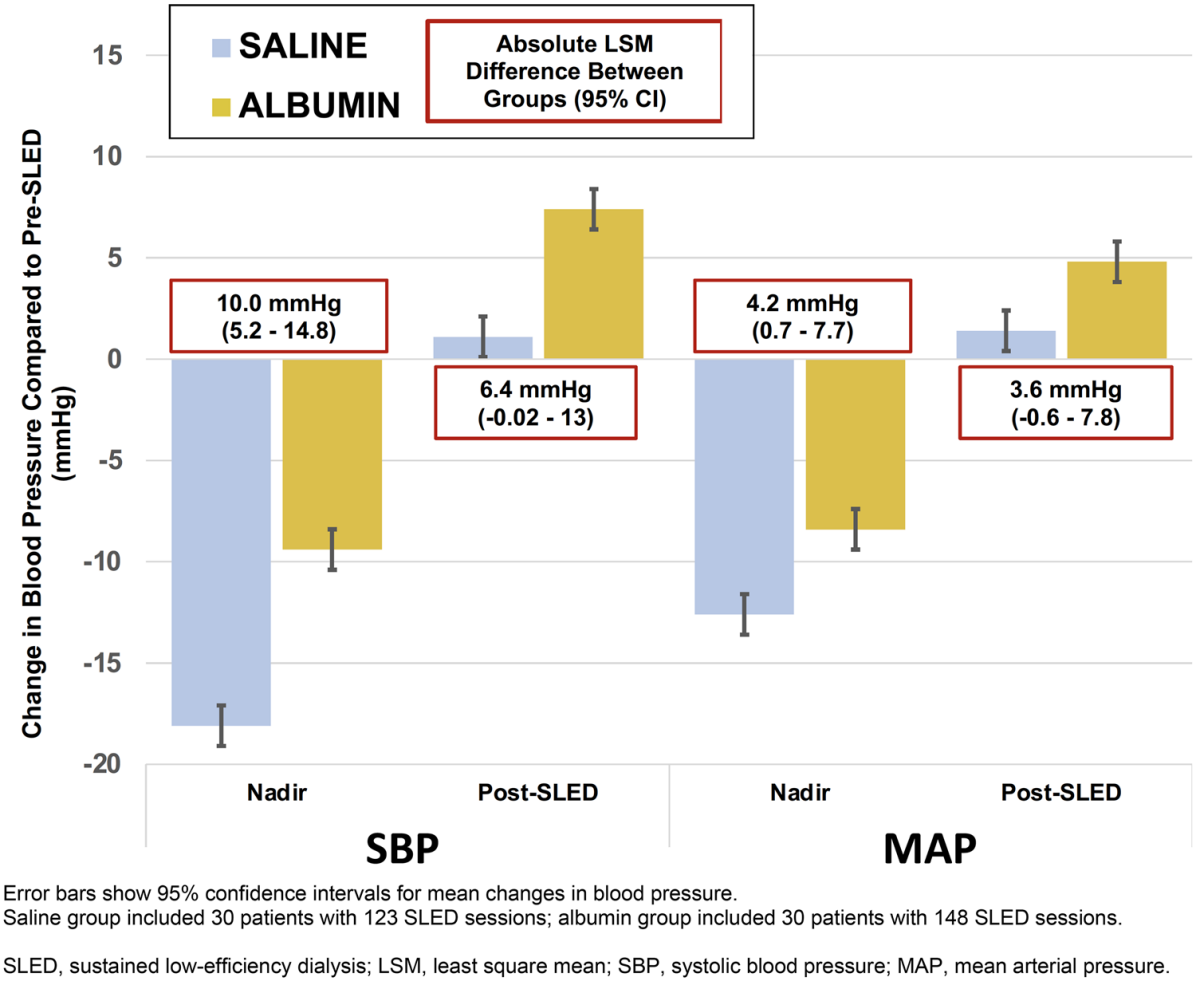
Unadjusted mean blood pressure changes (intra-SLED nadir and post-SLED compared to pre-SLED), and the modeled absolute difference between saline and albumin groups. Error bars show 95% confidence intervals for mean changes in blood pressure. Saline group included 30 patients with 123 SLED sessions; albumin group included 30 patients with 148 SLED sessions. SLED: sustained low-efficiency dialysis; LSM: least square mean; SBP: systolic blood pressure; MAP: mean arterial pressure

A drop in SBP >  = 20 mmHg occurred during 39% (48/123) of SLED sessions for the saline group and during 25% (37/148) of SLED sessions in the albumin group. Compared to the saline group, participants in the albumin group experienced a smaller drop in systolic blood pressure during SLED treatments across all definitions of hypotension assessed. This includes the maximal drop in SBP from start of treatment to the nadir intra-treatment value: mean difference 10.0 mmHg (95% confidence interval 5.2–14.8 mmHg). Mean changes in blood pressure (intra-SLED nadir blood pressures and post-SLED blood pressures relative to pre-SLED blood pressures) and the differences in those changes between the groups are shown in Fig. 2.

The need to start or increase the dose of vasopressors at any time during SLED sessions was not statistically different for the saline and albumin groups (24% (30/123) and 27% (40/148) of SLED sessions, respectively). Blood pressures and vasopressor use before, during and after SLED sessions are summarized in Table 3. Additional details regarding hemodynamic changes and vasopressor use during sessions are reported in the Additional file 1: Table S2.

**Table 3:** Hemodynamics during SLED sessions

Group	SBP*, Mean (SD)			MAP*, Mean (SD)			Vasopressor use, *n* (%)		
	Pre-SLED	Nadir	Post-SLED	Pre-SLED	Nadir	Post-SLED	Pre-SLED	Start/increase during SLED	Post-SLED
Saline *n* = 123^†^	118 (21)	99 (18)	119 (20)	78 (13)	65 (11)	79 (12)	99 (80)	30 (24)	95 (77)
Albumin *n* = 148^†^	116 (20)	107 (18)	124 (20)	78 (12)	69 (11)	83 (13)	91 (61)	41 (28)	80 (54)

SBP: systolic blood pressure; MAP: mean arterial pressure; SLED: sustained low-efficiency dialysis

*in mmHg

^†^Sessions

Patient outcomes are reported in Additional file 1: Table S3. Sixteen of 30 patients in the saline group (53%) died in the ICU compared to 11 of 30 (37%) in the albumin group; in-hospital mortality was 19 (63%) and 11 (37%), respectively. The median length of ICU and hospital stay did not differ significantly between groups Additional file 1: Table S3. Dialysis-dependence at the time of hospital discharge occurred in 2 of the 11 patients (18%) that survived to hospital discharge in the saline group and 5 of the 19 participants (26%) that survived to hospital discharge in the albumin group.

Additional file 1: Table S4 reports the analysis of fluid removal outcomes and blood pressure changes restricted to only the first SLED treatments that patients received (*N* = 60).

## Discussion

This single-center feasibility RCT sought primarily to assess the feasibility of a future larger clinical trial that will examine the efficacy of administering intravenous hyperoncotic albumin during SLED sessions to mitigate HIRRT and impact clinically meaningful outcomes. The number of refusals to participate was lower than expected and suggests that there may be fewer objections to participation in a study that involves an intervention that is already frequently applied in the care of critically ill patients and is known to be safe in that context [18]. The use of deferred consent in circumstances in which a surrogate decision-maker could not be reached prior to patients receiving their first SLED session ensured that fewer patients were missed for inclusion than would have been otherwise [19]. Adherence to the protocol was acceptable, meeting the pre-specified target of patients correctly receiving both doses of the assigned intervention (albumin or saline placebo) for at least 90% of SLED sessions overall. The rate of contamination (i.e., open label albumin use was low) but does suggest that a future study should consider refining inclusion criteria or restricting open label albumin use so as to reduce the likelihood of contamination. The pre-specified progression criteria were all achieved and this suggests that a larger trial powered for important clinical endpoints is feasible in this patient population and clinical setting.

While the principal objective of this pilot was to determine feasibility, we observed a number of differences between the groups with respect to blood pressure changes that support the hypothesis that albumin may reduce the incidence of hemodynamic instability among critically ill patients on RRT. Across all completed SLED sessions, albumin administration was associated with 10 mmHg less drop in SBP, on average, compared to saline. This did not appear to have an impact on ultrafiltration volumes, which were not different between the two groups. Notably, there were important baseline differences that could partially account for the hemodynamic findings. In particular, there was more vasopressor use at the start of treatment for patients in the saline arm (80% vs 61%).

Despite evidence of widespread use of hyperoncotic albumin for the prevention or treatment of hemodynamic instability during RRT, few trials have directly addressed its efficacy [10, 20]. Our exploratory hemodynamic findings are consistent with the results of a recent cross-over trial assessing the use of hyperoncotic albumin in hospitalized patients receiving hemodialysis (*n* = 65; 249 sessions) that included some sessions in ICU and also suggested that hyperoncotic albumin enhanced ultrafiltration [11]. To our knowledge, the only other trial to assess the use of hyperoncotic albumin in critically ill patients with AKI was an 8 person cross-over study that showed improved hemodynamics if 17.5% albumin was used as the priming solution for hemodialysis in septic patients with AKI [13]. Our trial suggests that hyperoncotic albumin may have a significant hemodynamic benefit even in critically ill patients with a high severity of illness on vasopressors and with rapid loss of intravascular albumin via damaged endothelial glycocalyx [10]. Given that it showed benefit in SLED, where ultrafiltration rates are relatively low compared to intermittent hemodialysis, it suggests that, even in patients receiving CRRT, administration of intravenous albumin might potentially improve hemodynamic tolerance of fluid removal.

We observed that significantly fewer patients died in hospital in the albumin group. While we feel that this finding warrants follow-up with a larger RCT, this study’s purpose was to evaluate feasibility and it was not powered to evaluate this outcome. In addition, the observed risk difference is too large to be plausibly explained by the intervention and the relatively small differences in hemodynamic instability that we observed.

There are several strengths of this trial. It was conducted and analyzed in accordance with a previously published protocol and data collection was detailed, including sufficient measures of illness severity, serum albumin, vasopressor use and fluid removal parameters that the observed intra-dialytic blood pressure differences in both study groups could be evaluated within a clear context and according to definitions defined a priori. Another strength is that broad inclusion criteria and the approved use of deferred consent facilitated a higher-than-anticipated recruitment rate. Although adherence was only 90% to the study assignment (lower than expected given that it was carried out by ICU nurses), we determined that the cause of some missed doses was not related to the trial conduct per se but due to the unique circumstance of implementing a new electronic medical record (plus computer order entry) at our institution in June 2019.

There are multiple important limitations of this study. While treatment allocation was blinded, most nurses would likely have been able to determine it since 25% albumin and saline are visually distinguishable when connecting tubing for infusion. Treating physicians likely remained blinded throughout although we did not formally assess this. Another theoretical limitation relates to the use of saline as a placebo in patients who may already be fluid overloaded and require ultrafiltration with RRT. Administration of additional fluid could be harmful as fluid overload is associated with worse outcomes in patients who require RRT [2, 21]. Practically, this is not a major concern: the overall volume of study fluid administered per session is small (200 mL) and is accounted for within the net ultrafiltration goal ordered for each patient’s SLED sessions. Notably, ordered ultrafiltration was achieved for nearly all sessions in both arms of the study suggesting that this intervention (or the placebo) did not contribute to worsening fluid overload. Another limitation relates to the generalizability of our findings. Our study consisted mainly of patients with septic shock. While the patients were enrolled at mixed medical–surgical ICUs at both campuses (with no cardiac surgery patients at either), no post-operative patients were included. Lastly, as a single-center study with a small sample size, as mentioned above with respect to vasopressor use prior to SLED treatments, baseline imbalances in the study groups were present.

## Conclusions

In conclusion, this single-center RCT of intravenous hyperoncotic albumin versus saline during SLED treatments in ICU achieved all of its predefined feasibility outcomes, thereby demonstrating that a larger scale trial is feasible. Future trials, adequately powered for outcomes, are needed to determine whether intravenous hyperoncotic albumin has a beneficial impact on HIRRT and relevant clinical outcomes for critically ill patients with AKI requiring RRT.

## Supplementary Information

**Additional file 1: Table S1.** Fluid removal during SLED sessions. **Table S2.** Additional hemodynamic outcomes. **Table S3.** Unadjusted mortality and length-of-stay. **Table S4.** Analysis of first SLED runs only. **File S1.** CONSORT 2010 checklist (pilot or feasibility trial).** File S2.** SAFER-SLED Logo.

## Data Availability

The complete anonymized dataset is available upon reasonable request with OHSN-REB approval.

## Abbreviations

RRT: Renal replacement therapy
SLED: Sustained low-efficiency dialysis
ICU: Intensive care unit
HIRRT: Hemodynamic instability during renal replacement therapy
AKI: Acute kidney injury
RCT: Randomized controlled trial
CRRT: Continuous renal replacement therapy
SBP: Systolic blood pressure
CI: Confidence intervals
