# Supplementary material for: Intravenous albumin for the prevention of hemodynamic instability during sustained low-efficiency dialysis: a randomized controlled feasibility trial (The SAFER-SLED Study)
Source: Ann Intensive Care. 2021 Dec 13;11:174. doi: 10.1186/s13613-021-00962-x (PMC8669086; doi:10.1186/s13613-021-00962-x)
Supplement: Supplementary file 1 — Additional file 1: Table S1. Fluid removal during SLED sessions. Table S2. Additional hemodynamic outcomes. Table S3. Unadjusted mortality and length-of-stay. Table S4. Analysis of first SLED runs only. File S1. CONSORT 2010 checklist (pilot or feasibility trial). File S2. SAFER-SLED Logo. [file 13613_2021_962_MOESM1_ESM.docx]

**ADDITIONAL FILE**

**Table of Contents**

**TABLE S1: Fluid Removal During SLED Sessions……………..…….……S2**

**TABLE S2. Additional Hemodynamic Outcomes………………..………....S2**

**TABLE S3. Unadjusted Mortality and Length-of-Stay……..………………S3**

**TABLE S4. Analysis of First SLED Runs Only………………………...……S3**

**FILE S1: CONSORT 2010 checklist (pilot or feasibility trial)……………..S4**

**FILE S2: SAFER-SLED Logo………………………………………………...…S8**

**TABLE S1. Fluid removal during SLED sessions**

| **Fluid removal parameter** | **Saline**  n=123*  Median (IQR) | **Albumin**  n=148*  Median (IQR) |
| --- | --- | --- |
| UF Goal (mL) | 3000 (2000, 4000) | 3500 (2500, 4000) |
| Actual UF (mL) | 2500 (1760, 3480) | 3000 (2000, 3980) |
| Percentage Achieved UF^†^  [Sessions excluded if Ordered UF = 0 mL]^‡^ | 99.7% (83.3, 100) | 99.8% (87.5, 100) |
| Percentage Achieved UF^†^ [Counted as 100% if Ordered UF = 0 mL] | 100% (94.3, 100) | 100% (94.2, 100) |
| Target UF – Achieved UF (mL) | 10 (0, 213)  [Mean (SD): 276 (812)] | 5 (0, 190)  [Mean (SD): 308 (833)] |

SLED, sustained low-efficiency dialysis; UF, ultrafiltration.

*sessions

^†^Calculated as: [Actual UF / UF Goal] X 100%

^‡^n=5 sessions for Saline group and n=6 sessions for Albumin group were excluded.

**TABLE S2. Additional hemodynamic outcomes**

| **Definition of Hypotension, n (%)** | **Saline**  n=123 treatments | **Albumin**  n=148 treatments |
| --- | --- | --- |
| Intra-SLED Nadir SBP <90 mmHg | 32 (26) | 16 (11) |
| Intra-SLED Nadir SBP <100 mmHg | 69 (56) | 51 (34) |
| Pre-SLED SBP – Intra-SLED Nadir SBP >=20 mmHg | 48 (39) | 37 (25) |
| Pre-SLED SBP – Intra-SLED Nadir SBP >= 30 mmHg | 25 (20) | 14 (9) |
| **Any Vasopressor Use, n (%)** | | |
| Pre-SLED session (start of treatment) | 99 (80) | 91 (61) |
| 2 hours into session | 92 (75) | 89 (60) |
| 4 hours into session | 78 (63) | 68 (46) |
| 6 hours into session | 88 (72) | 77 (52) |
| 8 hours into session (end of treatment) | 95 (77) | 80 (54) |
| **Cumulative Vasopressor Dose in Norepinephrine Equivalents*, Median (IQR)** | | |
| 0 hours into session (start of treatment) | 10 (4, 23) | 5 (2, 10) |
| 2 hours into session | 10 (4, 22) | 5 (2, 10) |
| 4 hours into session | 8 (4, 14) | 6 (3, 8) |
| 6 hours into session | 10 (4, 20) | 5 (2, 9) |
| 8 hours into session (end of treatment) | 10 (4, 20) | 4 (2, 8) |

*norepinephrine equivalents = [norepinephrine (mcg/min)] + [dopamine (mcg/kg/min) ÷ 2] + [epinephrine (mcg/min)] + [phenylephrine (mcg/min) ÷ 10] + [vasopressin (units/h) × 8.33]; includes only patients with any vasopressor use as per the section reporting ‘Any Vasopressor Use’.

SLED, sustained low-efficiency dialysis; SBP, systolic blood pressure; MAP, mean arterial pressure.

**TABLE S3. Unadjusted mortality and length-of-stay**

| **Outcomes** | **Saline**  (n = 30) | **Albumin** (n=30) | **Risk Difference**  % (95% CI) |
| --- | --- | --- | --- |
| Death in ICU, n (%) | 16 (53.3) | 11 (36.7) | 16.7 (-8.2, 41.5) |
| Death in hospital, n (%) | 19 (63.3) | 11 (36.7) | 26.7 (2.3, 51.1) |
| Average ICU length of stay in days, Median (IQR) | 10 (4,15) | 12.5 (8, 21) | --- |
| Average hospital length of stay in days, Median (IQR) | 23 (10, 57) | 31.5 (19, 55) | --- |

**TABLE S4. Analysis Restricted to First SLED Treatments**

| **Fluid removal parameter** | **Saline**  n=30  Median (IQR) | **Albumin**  n=30  Median (IQR) |  |
| --- | --- | --- | --- |
| Percentage Achieved UF^†^  [Sessions excluded if Ordered UF = 0 mL]^‡^ | 99.7 (0, 100) | 99.5 (17.8, 100) |  |
| Percentage Achieved UF^†^ [Counted as 100% if Ordered UF = 0 mL] | 100 (99.7, 100) | 100 (86, 100) |  |
| Target UF – Achieved UF (mL) | 0 (0, 100) | 10 (0, 90) |  |
| **Blood pressure changes** | **Mean (SD)** | **Mean (SD)** | **Mean difference (95% CI)** |
| Pre-SLED SBP – intra-SLED nadir SBP | 13.8 (16.9) | 7.3 (14.8) | 6.5±15.9 (-1.7, 14.7) |
| Pre-SLED SBP – post-SLED SBP | -5.9 (26.0) | -10.6 (21.1) | 6.7±23.6 (-7.6, 16.9) |
| Pre-SLED MAP – intra-SLED nadir MAP | 9.8 (9.7) | 6.5 (10.0) | 3.3±9.9 (-1.8, 8.4) |
| Pre-SLED MAP – post SLED MAP | -3.8 (14.1) | -6.2 (12.8) | 2.3±13.5 (-4.6, 9.3) |

SLED, sustained low-efficiency dialysis; UF, ultrafiltration.

^†^Calculated as: [Actual UF / UF Goal] X 100%

^‡^n=27 for both groups for this outcome only (3 patients in each group excluded for UF order of 0)

FILE S1. CONSORT 2010 checklist (pilot or feasibility trial)


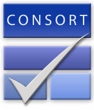
CONSORT 2010 checklist of information to include when reporting a pilot or feasibility trial*

| Section/Topic | Item No | Checklist item | Reported on page No |
| --- | --- | --- | --- |
| Title and abstract | | | |
|  | 1a | Identification as a pilot or feasibility randomised trial in the title | 1 |
|  | 1b | Structured summary of pilot trial design, methods, results, and conclusions (for specific guidance see CONSORT abstract extension for pilot trials) | 2 |
| Introduction | | | |
| Background and objectives | 2a | Scientific background and explanation of rationale for future definitive trial, and reasons for randomised pilot trial | 3 |
|  | 2b | Specific objectives or research questions for pilot trial | 3 |
| Methods | | | |
| Trial design | 3a | Description of pilot trial design (such as parallel, factorial) including allocation ratio | 3, 4 and previously published protocol^†^ |
|  | 3b | Important changes to methods after pilot trial commencement (such as eligibility criteria), with reasons | 4 |
| Participants | 4a | Eligibility criteria for participants | 3, 4 and previously published protocol^†^ |
|  | 4b | Settings and locations where the data were collected | 4 |
|  | 4c | How participants were identified and consented | 4 and previously published protocol^†^ |
| Interventions | 5 | The interventions for each group with sufficient details to allow replication, including how and when they were actually administered | 5 and previously published protocol^†^ |
| Outcomes | 6a | Completely defined prespecified assessments or measurements to address each pilot trial objective specified in 2b, including how and when they were assessed | 5, 6 and previously published protocol^†^ |
|  | 6b | Any changes to pilot trial assessments or measurements after the pilot trial commenced, with reasons | N/A |
|  | 6c | If applicable, prespecified criteria used to judge whether, or how, to proceed with future definitive trial | 5 |
| Sample size | 7a | Rationale for numbers in the pilot trial | Previously published protocol^†^ |
|  | 7b | When applicable, explanation of any interim analyses and stopping guidelines | N/A |
| Randomisation: |  |  |  |
| Sequence  generation | 8a | Method used to generate the random allocation sequence | Previously published protocol^†^ |
|  | 8b | Type of randomisation(s); details of any restriction (such as blocking and block size) | Previously published protocol^†^ |
| Allocation  concealment  mechanism | 9 | Mechanism used to implement the random allocation sequence (such as sequentially numbered containers), describing any steps taken to conceal the sequence until interventions were assigned | Previously published protocol^†^ |
| Implementation | 10 | Who generated the random allocation sequence, who enrolled participants, and who assigned participants to interventions | Previously published protocol^†^ |
| Blinding | 11a | If done, who was blinded after assignment to interventions (for example, participants, care providers, those assessing outcomes) and how | Previously published protocol^†^ |
|  | 11b | If relevant, description of the similarity of interventions | Previously published protocol^†^ |
| Statistical methods | 12 | Methods used to address each pilot trial objective whether qualitative or quantitative | 6 |
| Results | | | |
| Participant flow (a diagram is strongly recommended) | 13a | For each group, the numbers of participants who were approached and/or assessed for eligibility, randomly assigned, received intended treatment, and were assessed for each objective | 16 |
|  | 13b | For each group, losses and exclusions after randomisation, together with reasons | 16 |
| Recruitment | 14a | Dates defining the periods of recruitment and follow-up | 7 |
|  | 14b | Why the pilot trial ended or was stopped | 7 |
| Baseline data | 15 | A table showing baseline demographic and clinical characteristics for each group | 18 |
| Numbers analysed | 16 | For each objective, number of participants (denominator) included in each analysis. If relevant, these numbers  should be by randomised group | 18, 20 |
| Outcomes and estimation | 17 | For each objective, results including expressions of uncertainty (such as 95% confidence interval) for any  estimates. If relevant, these results should be by randomised group | 18, 20 |
| Ancillary analyses | 18 | Results of any other analyses performed that could be used to inform the future definitive trial | S2, S3 |
| Harms | 19 | All important harms or unintended effects in each group (for specific guidance see CONSORT for harms) | 8 |
|  | 19a | If relevant, other important unintended consequences | N/A |
| Discussion | | | |
| Limitations | 20 | Pilot trial limitations, addressing sources of potential bias and remaining uncertainty about feasibility | 11, 12 |
| Generalisability | 21 | Generalisability (applicability) of pilot trial methods and findings to future definitive trial and other studies | 10 |
| Interpretation | 22 | Interpretation consistent with pilot trial objectives and findings, balancing potential benefits and harms, and  considering other relevant evidence | 10-12 |
|  | 22a | Implications for progression from pilot to future definitive trial, including any proposed amendments | 10, 13 |
| Other information | | |  |
| Registration | 23 | Registration number for pilot trial and name of trial registry | 2, 4 |
| Protocol | 24 | Where the pilot trial protocol can be accessed, if available | 4 (previously published protocol^†^) |
| Funding | 25 | Sources of funding and other support (such as supply of drugs), role of funders | 14 |
|  | 26 | Ethical approval or approval by research review committee, confirmed with reference number | 4 |

Citation: Eldridge SM, Chan CL, Campbell MJ, Bond CM, Hopewell S, Thabane L, et al. CONSORT 2010 statement: extension to randomised pilot and feasibility trials. BMJ. 2016;355.

*We strongly recommend reading this statement in conjunction with the CONSORT 2010, extension to randomised pilot and feasibility trials, Explanation and Elaboration for important clarifications on all the items. If relevant, we also recommend reading CONSORT extensions for cluster randomised trials, non-inferiority and equivalence trials, non-pharmacological treatments, herbal interventions, and pragmatic trials. Additional extensions are forthcoming: for those and for up to date references relevant to this checklist, see [www.consort-statement.org](http://www.consort-statement.org).

^†^ Clark EG, McIntyre L, Ramsay T, Tinmouth A, Knoll G, Brown PA, Watpool I, Porteous R, Montroy K, Harris S, Kong J, Hiremath S. Saline versus albumin fluid for extracorporeal removal with slow low-efficiency dialysis (SAFER-SLED): study protocol for a pilot trial. *Pilot Feasibility Stud* 2019; 5: 72.


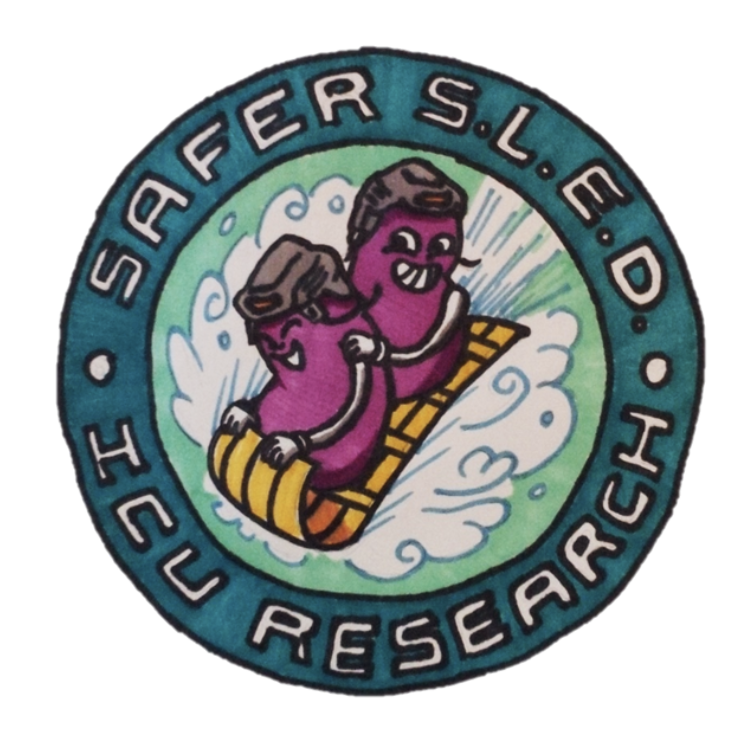
**FILE S2. SAFER-SLED logo**

Rebecca Porteus, 2018
